# Young children spontaneously invent wild great apes’ tool-use behaviours

**DOI:** 10.1098/rspb.2015.2402

**Published:** 2016-02-24

**Authors:** E. Reindl, S. R. Beck, I. A. Apperly, C. Tennie

**Affiliations:** School of Psychology, University of Birmingham, Edgbaston, Birmingham B15 2TT, UK

**Keywords:** tool use, problem solving, physical cognition, cognitive development, innovation, zone of latent solutions

## Abstract

The variety and complexity of human-made tools are unique in the animal kingdom. Research investigating why human tool use is special has focused on the role of social learning: while non-human great apes acquire tool-use behaviours mostly by individual (re-)inventions, modern humans use imitation and teaching to accumulate innovations over time. However, little is known about tool-use behaviours that humans can invent individually, i.e. without cultural knowledge. We presented 2- to 3.5-year-old children with 12 problem-solving tasks based on tool-use behaviours shown by great apes. Spontaneous tool use was observed in 11 tasks. Additionally, tasks which occurred more frequently in wild great apes were also solved more frequently by human children. Our results demonstrate great similarity in the spontaneous tool-use abilities of human children and great apes, indicating that the physical cognition underlying tool use shows large overlaps across the great ape species. This suggests that humans are neither born with special physical cognition skills, nor that these skills have degraded due to our species’ long reliance of social learning in the tool-use domain.

## 1. Introduction

The ability to use tools, i.e. to employ ‘unattached or manipulable attached environmental object[s]’ [1, p. 5], is not restricted to humans. Chimpanzees and orangutans—two of our closest living relatives—possess multiple tool-use ‘traditions’, i.e. tool use whose occurrence cannot be explained solely by genetic and environmental factors, but which is also influenced by social learning [2]. Although these traditions are said to bear similarities to human culture [3], the range and complexity of human tools are unique. Exploring the reasons for this uniqueness, researchers have focused on the role of special types of social transmission [4–8]: as Vygotsky [9] argued, humans' capacity to imitate and teach others enabled them to acquire behaviours that they could not (yet) have invented on their own. Over historical time, this ability allowed humans to gradually accumulate design improvements in tools [4,6]. In contrast, the evidence for imitation and teaching in great apes is weak ([5,10–12], but see [13]), so that tool use in these species is unlikely to be acquired via these mechanisms. The myriad of human tool forms thus represents the current end result of cumulative cultural evolution. This, however, begs the question as to what types of tool-use behaviours humans can invent without cultural learning. In other words, what are the roots of our tool cultures—both phylogenetically and ontogenetically?

This study aimed to explore this ‘baseline’ of human tool-use abilities by asking which tool-use behaviours human children would be able to invent on their own, i.e. without cultural resources such as instructions, demonstrations or eavesdropping. In order to determine whether a given tool-use behaviour can be invented individually, culturally naive individuals can be tested for spontaneous re-inventions of the behaviour [14]. Whereas for non-human animals, this can be done with captive individuals that happen to be naive to the behaviour in question [15], the case is more challenging for humans, as children learn how to use cultural tools from an early age [16,17]. By designing novel tasks, which children are unlikely to have encountered before, researchers can limit human participants' likelihood of drawing analogies from their previous cultural experience when solving the task. Therefore, we presented children with novel games and unusual apparatuses to ensure that they would need to solve the tasks via spontaneous individual inventions.

In order to identify candidate ecologically valid tool-using behaviours that may be invented spontaneously by children, we based our tasks on cultural tool-use behaviours currently observed in wild chimpanzees [3] and orangutans [18]. This approach also allows comparisons of spontaneous tool-use abilities across species. This is important as one cannot *a priori* assume that humans possess equal, worse or better basic tool-use skills than great apes—this must be tested explicitly [19]. The ability to use tools flexibly is thought to require a range of physical cognition capabilities. First, flexible tool users (of any given species) need to be capable of realizing that objects can be used as tools [20], of recognizing the tools' functionality [21], and of causal reasoning [20]. In addition, more general abilities have been proposed to be required, such as recombining information in order to solve novel tasks [22] and learning from perceptual-motor feedback [20]. Finally, flexible tool use is thought to also depend on a propensity for object manipulation [22], an enhanced working memory capacity to process the increased problem–solution distance of tool-use tasks [20], and the ability to inhibit and switch between strategies.

Deaner *et al*. [23] found that some non-human primates possess better domain-general cognition abilities than others and thus speculated that these differences in general intelligence might account for differences in these species' tool-use abilities. From this, one might expect human superiority to other primates on tool-use tasks. In contrast, Ruiz & Santos [21] argue that humans and great apes possess a similar understanding of the physical and functional aspects involved in tool use. Another approach might expect human tool-use abilities to be impoverished compared to other species: modern human intelligence is argued to be based on our species-unique cultural learning mechanisms [4,6,7]. Such a reliance of human intelligence on social transmission could have led to the loss of some individual cognitive skills. Our species' cultural intelligence could have made individual ‘baseline’ physical cognition obsolete.

One previous study [19] used a battery of similar tasks for great apes and human children to compare the cognitive skills of these species, and while it suggested that children had more advanced social skills than great apes, no differences were found in the physical cognition abilities of children and chimpanzees (with orangutans performing slightly worse). However, the tasks used in this study were solely inspired by human behaviour and lacked ecological validity from the perspective of the great apes—which are arguably closer to the state of our common ancestor than modern humans [24]. Thus, using great ape behaviours as the basis for tasks might represent a phylogenetically more appropriate approach if we aim to make inferences about our last common ancestor's cognitive capacities. Our approach, by creating tasks for humans based on great ape behaviours, now complements Herrmann *et al*.'s [19] method, and in combination both represent a more valid approach to the comparative analysis of human physical cognition.

The first aim of this study was to investigate whether children between 2 and 3.5 years would be able to spontaneously invent tool-use behaviours required to solve naturally occurring problems that wild great apes solve. For this, we created the Great Ape Tool Test Battery (GATTeB), containing 12 tasks derived from cultural tool behaviours observed in wild chimpanzees and/or orangutans. To be able to conclude that a behaviour lies within the spontaneous cognitive capacities of children, we would need to observe its spontaneous invention in at least two participants [25] (positive evidence from a single child was judged to be insufficient as the observed action could have been produced by chance; see also electronic supplementary material).

If we found an overlap in the spontaneous tool behaviours of children and great apes, this would suggest, from a *phylogenetic* perspective, that these behaviours were also likely within the cognitive capability of our last common ancestor. With regard to the *ontogenetic* roots of human tool use, knowledge about spontaneous tool behaviours in children is generally sparse. The ability to spontaneously use tools (such as sticks or cloths) to obtain out-of-reach objects has been shown to develop between 8 and 24 months of age [26]. However, whether young children are also able to spontaneously use tools for other purposes, such as extracting or perforating objects, is still unknown. Thus, our findings contribute to the developmental literature insights into whether children would also be able to spontaneously use tools for these purposes that have not been investigated, and which are ecologically highly relevant.

Our second aim was to investigate whether tool-use behaviours which appear to be more difficult to invent for wild great apes would also be more challenging for children to invent spontaneously. Cultural tool behaviours in wild great apes differ with regard to their observed frequency: while some behaviours are shared by several communities within a species, e.g. termite-fishing in chimpanzees, others are less frequent, e.g. algae scooping [3]. The behaviours' observed frequency is thought to represent their ease of individual invention [27]. Thus, the tool behaviours occurring rarely in wild great apes may pose greater demands on cognitive abilities (e.g. planning, working memory) and/or motor skills (e.g. physical strength, fine-motor skills) when compared to more frequent tool behaviours. Would behaviours that appear to be hard to invent for wild great apes also be more difficult to invent for children in our study? To investigate this, we divided the 12 GATTeB tasks into two groups (*low-frequency* and *high-frequency*), according to the frequency with which the respective great ape behaviours were observed in the wild.

We studied children from 2 to 3.5 years of age. We chose the lower end of our age range to be 2 years as Herrmann *et al*. [19] have claimed that 2-year-olds represent a meaningful point of comparison with great apes. However, we chose a broader age range than [19] for two reasons: first, our tasks represent more challenging tool problems, with many of them posing additional demands on children's planning, fine-motor skills or physical strength. Second, by allowing for variation in participants' age we were able to examine whether any difference between low- and high-frequency tasks was stable over developmental time.

## 2. Material and methods

### (a) Creation of the GATTeB

We based our test battery on tool-use behaviours described in the current reviews of potentially cultural behaviours in wild chimpanzees [3,11,28,29] and orangutans [18,30]. Where viable, we transferred cultural tool-use behaviours to problem-solving games for human children (for the selection process, see the electronic supplementary material, table S1), resulting in 12 novel tasks (table 1; for pictures, see electronic supplementary material, figure S1). Although the great ape tool behaviours are all exhibited within a foraging context, we did not opt to use food as a reward for human participants due to ethical issues. Instead, each task was designed as a game in which children could win a sticker. Stickers represent a highly valuable and desirable good for most Western children throughout the preschool age—and are thus motivating for children.

**Table 1.** Selected great ape tool-use behaviours and description of the GATTeB tasks.

behaviour (frequency)	description of great ape behaviour	description of task	allocated testing time
insect-pound (low)	use stick to pound bottom of hole to break and retrieve insects	use stick to retrieve Play Doh^®^ balls from tube by prodding them	2 min
perforate (low)	use stick to make probing holes in termite nests	use stick to perforate barrier in box to retrieve sticker	2 min
nuthammer (low)	use piece of wood/stone to crack nuts	use clay hammer to crack plastic nut to obtain sticker	2 min
algae scoop (low)	use twig to scoop for algae on water surface	use stick to scoop for strip of plastic in polystyrene beads to obtain sticker	2 min
ground puncture (low)	use stout stick to puncture underground insect nest	use stout stick to puncture layer of Plasticine in box to retrieve sticker	3 min
seed extraction/nut extract (low)	use twig to extract seeds from nut/fruit	use stick to extract pom poms from box	2 min
marrow pick (high)	use small stick to retrieve marrow of long bones	use stick to retrieve sponge attached to sticker from tube	1 min
fluid dip (high)	use sticks to fish for honey or water	use stick to dip for paint in tube	1 min
ant-dip-wipe (high)	use stick to collect ants, then wipe off and eat	use wet stick to collect polystyrene beads, then wipe off into box	3 min
termite-fish leaf-midrib (high)	use leaf-midrib to retrieve termites from nest	subtract paper ‘leaf’ from stick and use stick with Velcro^®^ at ends to fish for scourer pieces in box	2 min
lever open/stick as chisel (high)	use stick as lever to enlarge insect nest entrance in log or ground	use stick as lever to enlarge hole in Plasticine lid of a mug to retrieve ball with sticker attached to it	1 min
termite-fish/tree-hole tool-use (high)	use stick to extract insects from nest	use stick with Velcro^®^ at ends to fish for scourer pieces in box	1 min

The GATTeB tasks were divided into two groups, according to the frequency with which the respective great ape behaviours were reported to appear in the wild (electronic supplementary material, table S2): behaviours described as customary (i.e. occurring in (almost) all members of at least one age-sex class), habitual (observed repeatedly in more than one individual, but not customary) or present (clearly identified, but not customary/habitual [28]) in three or more distinct populations of a species were assigned to the *high-frequency* group; behaviours reported to be customary, habitual, present or rare (i.e. behaviour too rare to spread socially [18]) in two or fewer non-connected communities were assigned to the *low-frequency* group. Nine of the tasks were derived from behaviours only shown by chimpanzees. Three tasks (seed extraction/nut extract, termite-fish/tree-hole tool-use, lever open/stick as chisel) were based on behaviours that occur in a comparable fashion in both chimpanzees and orangutans, and which were thus combined.

### (b) Subjects

Fifty children (24 boys) between 26 and 41 months (mean age = 33.04 months, s.d. = 3.69 months; twelve 26- to 30-month-olds, thirty 31- to 36-month-olds, eight 37- to 41-month-olds) were recruited from a metropolitan area in the UK and a small town in Germany. Participants were tested individually by the same female experimenter in nurseries, a science museum and the university laboratory. Written informed consent was obtained by children's guardians prior to the study. Each participant was administered four tasks (two high- and two low-frequency tasks, randomly chosen and put in one of the following two orders: high-low-high-low or low-high-low-high). Each task was administered to between 15 and 17 children.

### (c) Procedure

A warm-up game in which children had to break wooden sticks familiarized children with the fact that they were allowed to modify and destroy material during the experimental session. This was important as several tasks required participants to break or perforate material and sometimes also involved applying physical force (e.g. ground puncture). The GATTeB tasks were designed to be solved by using a tool. Based on observations in a pilot study, children were given 1 min (termite-fish/tree-hole tool-use, lever open/stick as chisel, fluid dip and marrow pick, as children found the solutions quickly), 2 min (insect-pound, nuthammer, termite-fish leaf-midrib, perforate, algae scoop and seed extraction/nut extract) or 3 min (ground puncture (as children had to apply much physical force) and ant-dip-wipe (as children tended to only slowly approach the apparatus)) to solve the tasks. Children were rewarded with a sticker for each task, regardless of success.

### (d) Coding

Participants' behaviour was live-coded. We documented whether children picked the tool up or picked it up and used it in any way (tool pick up/use), whether they used the tool in a way that could *potentially* lead to success (correct tool use) and whether they succeeded on the task by using the tool in the correct way (correct success). The rare cases in which children succeeded in a non-intended way, i.e. without a tool, were scored as incorrect success and were excluded from the analysis. Data from eight children (16% of the sample; the only children for whom video material was available) was coded by a second rater blind to the hypotheses of the study. Cohen's *k* for tool pick up/use was perfect (*k* = 1.000), and excellent for correct tool use (*k* = 0.874) and correct success (*k* = 0.913).

## 3. Results

Fifty children completed a set of four tasks each, resulting in 200 trials of which 193 were valid. One trial had to be excluded because of an intervention of nursery staff, two trials due to experimenter error. Four trials were excluded after being scored as incorrect success.

Table 2 gives a detailed overview of children's rates of tool pick ups/uses, correct tool uses and correct successes. Out of the 193 times the tasks were conducted with children, the respective tools were picked up in 80.3% of the cases. Low- and high-frequency tasks did not differ with regard to their rates of tool pick up/use (see the electronic supplementary material). Thus, children were motivated to interact with the tools.

**Table 2.** Rates for tool pick up/use, correct tool use and correct success for low- and high-frequency tasks.

	frequency of corresponding great ape behaviour	task (*n*_valid trials_)	tool pick up/use (% of valid trials)	correct tool use (% of valid trials)	correct success (% of valid trials)
	low	IN (17)	17 (100%)	16 (94.1%)	4 (23.5%)
		PER (16)	11 (68.8%)	11 (68.8%)	1 (6.3%)
		NUT (15)	10 (66.7%)	1 (6.7%)	0 (0%)
		AE (15)	10 (66.7%)	9 (60.0%)	6 (40%)
		GR (15)	10 (66.7%)	8 (53.3%)	2 (13.3%)
		SEED (17)	15 (88.2%)	15 (88.2%)	5 (29.4%)
total_low_		95	73 (76.8%)	60 (63.2%)	18 (18.9%)
	high	MA (17)	13 (76.5%)	13 (76.5%)	3 (17.6%)
		FD (17)	15 (88.2%)	14 (82.4%)	14 (82.4%)
		ADW (17)	16 (94.1%)^a^	10 (58.8%)	10 (58.8%)
		TFLF (17)	15 (88.2%)	8 (47.0%)	6 (35.3%)
		LEV (15)	12 (80%)	12 (80%)	1 (6.7%)
		TF (15)	11 (73.3%)	11 (73.3%)	9 (60%)
total_high_		98	82 (83.7%)	68 (69.4%)	43 (43.9%)
*N*_total_		193	155 (80.3%)	128 (66.3%)	61 (31.6%)

^a^Note that children were explicitly told to pick up the tool.

Correct tool use was observed in 11 tasks (and more than eight times in each of them), indicating that the majority of the great ape tool solutions could be invented individually by human children. ‘nuthammer’ (i.e. hammering of a plastic nut with a clay hammer) was the only task in which only one child used the tool correctly. Since this child did not succeed in breaking the nut, and since no second child used the tool correctly, we cannot rule out the possibility that this instance of correct tool use was due to chance. Low- and high-frequency tasks did not differ with regard to their rates of correct tool use (see the electronic supplementary material).

In terms of correct success, we found that in 31.6% of the trials children solved the given task correctly. We also found a large numeric difference in correct success between low- and high-frequency tasks: whereas children solved 19% of the low-frequency trials, the success rate for high-frequency trials was at 44%. In order to investigate this difference statistically, we used generalized linear mixed models in R [31] with age, sex and frequency as fixed effects and a random intercept for subjects. As we found that sex did not contribute significantly to the model fit (see the electronic supplementary material), we dropped this variable from the final model. Results based on the final model revealed that, first of all, children's success was significantly related to age (*p* < 0.001). With each month increase in age, children were 1.3 (95% CI [1.1; 1.4]) times more likely to succeed. Independent of this age effect, we found that frequency of tool usage by wild great apes significantly predicted success in the human children (*p* < 0.001): compared to low-frequency tasks, tasks in the high-frequency group were 4.4 (95% CI [2.1, 9.1]) times more likely to be solved (electronic supplementary material, table S3). Thus on average, great ape low-frequency tasks were also low frequency for children and great ape high-frequency tasks were also high frequency for human children (note that not all tasks matched this pattern, see the electronic supplementary material).

We did not find an interaction between age and frequency, i.e. although older children were more successful than younger ones across all tasks, they were still experiencing the low-frequency tasks as more difficult than the high-frequency tasks. The frequency effect was thus stable over the age range. Whether this suggests that the frequency and age effects reflect distinctive or common underlying factors remains open to debate and is a focus for future studies.

In order to investigate whether children's success was affected by the tasks' allocation of differing amounts of times for their completion (1, 2 or 3 min), we reran the model and included a fixed effect for time. Results showed that time did not make any significant contribution in the model (*χ*^2^(1) = 0.560, *p* = 0.454).

## 4. Discussion

Our study found that the majority (at least 11 out of 12) of the investigated wild great ape tool-use behaviours are individually re-inventable by human children and that there is a close relationship between the difficulty level of these behaviours and individual discovery rates for both humans and great apes. Unlike a previous study—i.e. Herrmann *et al*. [19], whose tasks were biased towards the human case—we validated our tasks ecologically by basing them on great ape tool behaviours as described in the wild. Thus, our study presents phylogenetically more appropriate tasks for the study of the physical cognition of our last common ancestor.

Children showed spontaneous tool use in the majority of our tasks, suggesting that nearly all of the studied behaviours lie within the realm of what humans can invent without observing the solution or having it demonstrated. The large overlap between the behaviours that can be invented spontaneously by great apes and human children suggests that young children's physical cognition skills are at least on the same level as those of great apes. These findings do not rule out the possibility that there might be physical cognition tasks in which young children outperform great apes. However, in combination with the study by Herrmann *et al*. [19]—who presented great apes and 2-year-old human children with tasks based on human behaviours and who found no difference in the performance of great apes and children—the results of this study suggest that *ontogenetically*, humans do not seem to differ from great apes with regard to their baseline set of physical cognition abilities. From a *phylogenetic* perspective, humans' basic tool-use abilities do not appear to have become degraded by our species' long reliance on social learning and teaching. However, to eventually answer the question whether the physical cognition abilities of great apes (including humans) are comparable or whether humans possess enhanced physical cognition skills, future studies will need to present humans and great apes with tool tasks completely novel for both (e.g. tasks based on tool behaviours observed in other, non-primate species and which are not already known to be exhibited by great apes or children).

Going back to our results, we also found that children were more likely to solve tasks based on great ape behaviours which occur with high frequency in the wild compared with more low-frequency tasks, and this effect did not change with age. Thus, it seems that tool tasks in the low-frequency group possess features that make successful tool use more difficult for both humans and great apes, i.e. which make them more challenging for the evolved cognition of these species. A possible reason for the enhanced difficulty of low-frequency tool tasks might be that, whereas high-frequency behaviours mainly require the tool user to perceive and select the correct affordances, low-frequency behaviours may possess additional cognitive or non-cognitive demands. For example, some of the high-frequency behaviours may only require the insertion and subsequent retrieval of a stick into a hole (see, for example, termite-fish/tree-hole tool-use, fluid dip). In contrast, low-frequency tasks might pose additional demands on, for example planning (e.g. perforate, consisting of two steps: first breaking the barrier with the stick and then turning the box upside down to retrieve the sticker; similarly, chimpanzees need to first break the entrance to the termite mound with a stick and then use a different stick to retrieve the insects); fine-motor skills (e.g. in seed extraction/nut extract, the target objects have to be retrieved dextrously); physical strength (e.g. in ground puncture or nuthammer) or working memory (e.g. in nuthammer, tool users need to attend to several objects simultaneously). However, identifying the specific reasons for the difficulty of low-frequency behaviours will be the target of future studies.

Whereas low- and high-frequency tasks differed with regard to children's success rates, we found no effect of frequency on correct tool use. In both low- and high-frequency tasks, children were equally likely to show the correct tool behaviour, and did so in more than two-thirds of the trials. This finding underlines young children's proneness to use tools in meaningful ways to try to solve even novel problems. However, whether children's disposition to use tools is also followed by task success seems to depend on task type: in high-frequency tasks, both children's tool use and success rates were relatively high. In contrast, in low-frequency tasks, even though children were equally likely to use the tools correctly, tool use was less likely to result in success. This finding highlights that correct tool use does not necessarily imply task success. Other cognitive and/or non-cognitive demands have to be met so that correct tool use can be ‘translated’ into success. This ‘translation process’ seems to be more demanding for the low- compared to the high-frequency tasks (see above for a speculation about possible underlying reasons).

We also found that older children were more likely to solve the GATTeB tasks than younger children. This suggests a development between 2 and 3.5 years of age of capacities allowing children to more successfully meet the demands of the studied tool tasks. Future work will need to identify these capacities; potential candidates may be improvements in fine-motor skills, visual attention, working memory, physical strength and planning. However, we did not find an interaction between age and frequency. That is, even though the older children in our sample might have possessed better planning and fine-motor skills than younger children, this did not suffice to help the older children overcome the demands of the low-frequency tasks. Thus, we conclude that the frequency effect is stable across the studied age range.

It might be argued that our tasks were only based on wild tool cultures of two of the four currently living genera of great apes. However, wild gorillas and bonobos exhibit only very low levels of tool use in the wild and thus failed to provide the wild input for our tasks. Nonetheless, these genera readily use tools in captivity—i.e. when a need arises to do so [32,33]. Thus, while they did not contribute to our validated list of tasks, they are no exception from the line of widespread tool use across the great apes.

Our findings support the notion that the last common ancestor of humans and great apes—living approximately 14 Ma—was already capable of the tool-use behaviours studied here (and that they also found the low-frequency tasks more difficult to invent). These behaviours thus represent a phylogenetic basic state of human tool use—and they would not have required sophisticated cultural transmission mechanisms such as imitation and teaching. This situation matches very closely the current state of affairs of great ape tool cultures, which represent ‘latent solutions’ [27]: i.e. the range of tools inventable by individual great apes.

Our study gives a first insight into the ontogenetic and phylogenetic roots of human tool culture by identifying a range of ecologically relevant tool-use behaviours which human children—tested on tasks validated by great ape tool behaviours—can invent on their own. In conjunction with previous research [19], in which great apes solved tasks validated by modern human behaviour, we conclude—contra recent claims [34]—that in the tool-use domain humans are not born special.

## Supplementary Material

Reindl et al. ESM ‘clean’

## Data accessibility

The data reported in this study are available from the Dryad Digital Repository: http://dx.doi.org/10.5061/dryad.jg34q. R is a free software environment for statistical computing and graphics and can be downloaded from the R-project website: https://www.r-project.org/.
