# Supplementary material for: Young children spontaneously invent wild great apes’ tool-use behaviours
Source: Proc Biol Sci. 2016 Feb 24;283(1825):20152402. doi: 10.1098/rspb.2015.2402 (PMC4810823; doi:10.1098/rspb.2015.2402)
Supplement: Reindl et al. ESM ‘clean’ [file rspb20152402supp1.pdf]

Supplementary Information

This file includes:

Methods and Material, Tables S1, S2, Figure S1

Supplementary Data, Table S3

1. Methods and Material

Creation of the Great Ape Tool Test Battery (GATTeB)

The GATTeB was created based on reviews of potentially cultural behaviors observed in wild chimpanzees [3,11,28,29] and orangutans [18,30]. First, we extracted the behaviors from these reviews and identified 124 different behavioral patterns (Table S1). Behaviors that occurred in both chimpanzees and orangutans were counted only once (printed in bold). Behavioral variants describing similar tool-use actions and functions were merged: *Marrow pick*, *Eye eat*, and *Brain eat* (described in chimpanzees) were comprised to one behavioral variant labelled *Marrow pick. Termite fish*, *Ant-fish*, *Ant-dip,* and *Grub extraction* (chimpanzees) as well as *Tree-hole tool-use (*orangutans) were combined to *Termite fish/Tree-hole tool-use. Nut extract* (chimpanzees) and *Seed extraction* (orangutans) were combined to *Seed extraction/Nut extract.* Finally, *Branch drag/Drag branch* (chimpanzees) and *Branch dragging display on ground* (orangutans) were combined to *Branch drag*.

Table S1. Excluded behavioral variants in chimpanzees and orangutans and reasons for their exclusion.

Reason for exclusion	Behavioral pattern	
	Chimpanzees	Orangutans
	No tool use	*No tool use*
No tool use	1 Buttress-beat[a]	19 Water play[c]
	2 Branch-clasp[a]	20 Coercive hand-holding[c]
	3 Muzzle rub[b]	21 Throat scrape[c]
	4 Skull pound[b]	22 Twig biting[c,d]
	5 Driver ant-hand[b]	23 Symmetric scratch[c,d]
	6 Termite mound-pound[b]	24 Nest smack[c]
	7 Herbal pith[b]	25 Raspberry[c,d]
	8 Ground day-nest[b]	26 Snag riding[c,d]
	9 Ground-night-nest[a]	27 Drink from bottom of pitcher plant[c]
	10 Day cushion[b]	28 Slow loris eating[c,d]
	11 Day nest[b]	29 Dead twig sucking[c,d]
	12 Rain dance[a]	30 Nest destruction[d]
	13 Knuckle-knock[a]	31 Bouquet feeding[c,d]
	14 Hand-clasp[a]	32 Long-call vibrato[c]
	15 Index-hit[a]	33 Kiss-squeak with hands[d]
	16 Leaf-groom[a]	34 Copulation on female‘s nest[c]
	17 Food-pound onto wood[a]	35 Using Asplenium fern to rest or sleep in[c]
	18 Food-pound onto other[a]	36 Females rubbing their genitals together[c,d]
		37 Biting through vine to swing across gap[c]
		38 Biting through vine to release tree to sway to adjacent tree[c,d]
		39 Washing face and arms with water from tree hole[c]
		40 Male and female use the same nest to spend the entire night[c]
		41 Play nests[d]
		42 Nest as social refuge[c]
		43 Snag crashing[d]
		44 Sneaky nest approach[c,d]
		45 Hide under nest[c,d]
		46 Artistic pillows[d]
		47 Bridge nest[d]
		48 Carry leafy branch to different tree to build nest[d]
		49 Leaf bundle[c,d]
	Tool use	*Tool use*
Universal behaviors	50 Branch-shake[a]	
	51 Play-start[a]	
	**52 Drag branch/branch drag[a,b]**	
	53 Leaf sponge[a,b]	
	54 Investigatory probe/Inspect stick[a,b]	
Associative tool use	55 Sponge push-pull[a]	
	56 Anvil prop[a,b]	
	57 Open and probe[a,b]	

Hygiene behaviors	**58 Leaf-napkin**[a,b]	**58 Leaf napkin**[c,d]
	59 Comb[a]	61 Use leaf to clean body surface[c,d]
	60 Nasal probe[a]	62 Moss cleaning[c]
		63 Nail cleaning[c]
		64 Tooth cleaning[c]
		65 Tooth pick[c]
		66 Chewing leaves into pulp then smearing foam over body[c]
Handling ecto-parasites	67 Leaf-squash[a]	
	68 Leaf-inspect[a]	
Wound care	**69 Leaf-dab**[a,b]	**69 Poultice use**[c]
	70 Leaf wadge[b]	
	71 Wound inspect[b]	
Aggressive behavior	72 Club/stick club[a,b]	
	73 Aimed-throw/missile throw[a,b]	
	74 Flail twig[b]	
Sexual behavior		75 Autoerotic tool[c,d]
Communi-cative context	76 Leaf-clip, mouth[a]	**52 Branch dragging display on ground**[c,d]
	77 Leaf-clip, fingers[a]	83 Kiss-squeak with leaves[c,d]
	78 Leaf-strip[a]	84 Leaf-wipe in kiss-squeak context[d,c]
	79 Branch din[a]	85 Hiding behind detached branch from predators/humans[c,d]
	80 Branch-slap[a]	
	81 Shrub-bend[a]	
	82 Stem pull-through[a]	
Aims at provoking reactions from other animal species	**86 Fly-whisk**[a,b]	**86 Branch as swatter**[c,d]
	87 Bee probe[a,b]	
	88 Expel/stir[a]	
Spontane-ous behavior	89 Self-tickle[a,b]	90 Scratch stick[c,d]
Possible early cultural influence in humans	91 Seat stick[a]	93 Branch cushion[c]
	92 Stepping-stick[a]	94 Leaf gloves/cushions[c,d]
		95 Cover head with leafy branch/leaves against stinging bees[c]
Leaves/ twigs/other material not allowed to be taken to nurseries	96 Container[a]	103 Leaf scoop[c,d]
	97 Leaf-wipe[a,b]	104 Branch scoop[c.d]
	98 Leaf-brush[a]	105 Sponging[c,d]
	99 Leaf mop[a]	
	100 Seat vegetation[a]	
	101 Brush-stick[a]	

	102 Resin-pound[a]	
Potentially harmful to children		106 Use gloves to get into ants' nest or to handle spiny fruits[c]
Nest building behaviors		107 Bunk nests[c,d]
No clear behavioral description	108 Dig[a]	
Not transferable to laboratory setting	**109 Branch hook/hook stick[a,b]**<br>110 Leaf rain cover[b]<br>111 Pestle-pound[q,b]	**109 Branch hook[c]**<br>112 Sun cover[c,d]

Notes. [a]Behavior listed in Whiten et al. (1999, 2001) and Langergraber et al. (2011). [b]Behavior listed in Boesch, C. (2012). [c]Behavior listed in van Schaik et al. (2009). [d]Behavior listed in van Schaik et al. (2001). Behaviors in bold print are the same in both species and thus counted only once.

In a second step, we deleted behaviors not including tool use (n = 49). From the remaining 75 tool-use behaviors we excluded 5 behaviors classified as universals because they do not contain variance attributable to culture. We also excluded three behaviors involving the use of more than one tool, as our focus was on "simple" tool use.

We assessed each of the remaining behaviors with respect to transferability to problem-solving tasks for children. This meant that we had to deselect those that failed to transfer: We deselected nine behaviors related to great ape hygiene; two behaviors aiming at handling ectoparasites; three behaviors for wound care; three behaviors shown in an aggressive context; and one sexual behavior. Another 10 behaviors were excluded because they were used in a communicative context. A further three behaviors were deselected because they aimed at provoking reactions from other animal species, which was difficult to emulate in the lab: *Fly-whisk/Branch as swatter, Bee probe*, and *Expel/stir*. Two behaviors had to be excluded because they were dependent on rare and spontaneous incidents which were difficult to provoke in the laboratory (*Self-tickle* and *Scratch stick*). Four behaviors were deselected because we agreed that children are likely to possess cultural knowledge about them: *Seat stick* and *Branch cushion*

(cushions), *Stepping-stick* (shoes), and *Cover head with leafy branch to protect against stinging bees* (hats). Another 10 behaviors had to be excluded because we were not allowed to take leaves, twigs or other material into nurseries. *Use gloves to get into ants' nest or to handle spiny fruits* was deselected because it was regarded as potentially harmful to children. One orangutan behavior was excluded because it was related to nest building behavior *(Bunk nests). Dig* was deselected because we did not find a clear behavioral description in the primary sources; its differentiation from *Lever-open* seems to be unclear. Finally, four behaviors were dropped because we regarded them as not practicable in a laboratory study for the following reasons: *Branch hook/Hook stick*, because this task would have required providing an out-of-reach stick-like object that would possibly have to be attached at the ceiling. This task was regarded more suitable for a laboratory environment instead of nurseries. *Leaf rain cover* and *Sun cover*, because these actions are elicited by circumstances which cannot be controlled in an experimental setting (sun, rain). *Pestle-pound*, because a task in which children would have to destroy something by exerting much physical force with a large stout stick in several pounding actions would not meet the space and safety requirements in the nurseries.

The remaining 12 tasks formed the GATTeB and were divided up into two groups according to their observed frequency in the wild (Table S2). We decided to combine those behaviors which were classified by the reviews as 1) being a rarity [18] or 2) being present, customary or habitual [3] for no more than two great ape populations into a *low-frequency* group. The populations had to be separate, i.e. not be regarded as connected as it is the case, e.g. in the chimpanzee groups of Mahale (Mahale B, K, and M) or Taï (Taï North and South). To give an example, chimpanzees in Bossou as well as in both Taï groups have been observed to use stones or wooden clubs as hammers to crack open nuts. Since the two Taï groups experience exchange of group members, which allows the spread of cultural innovations, they are likely not independent of each other and we therefore considered them as a single population.

Consequently, Nuthammer was counted for two independent groups only (Bossou and Taï) and thereby falls into the *low-frequency* group. The *high-frequency* group consisted of behaviors which were present, customary or habitual in at least three distinct populations.

In terms of three tool-use behaviors that occurred in both chimpanzees and orangutans (Seed extraction/Nut extract, Lever open/Stick as chisel, and Termite fish//Tree-hole tool-use) we made the following decision: As Seed extraction and Nut extract were found to occur with low frequency in orangutans and chimpanzees, respectively, we assigned Seed extraction/Nut extract to the low-frequency group. For Lever open and Termite fish we found that these behaviors are highly frequent in chimpanzees, but the respective versions in orangutans are of only low frequency. We decided to list both behavioral variants as highly frequent, as they occurred with high frequency in at least one species.

Table S2. Classification of the tool-use behaviors based on their frequency in the wild.

Behavior	Frequency in the wild	Frequency group
Insect-pound (IN)[a]	Rarity (present in Bossou)	Low-frequency
Perforate (PER)[a,b]	Habitual/customary in one population (Goulaougo)	
Nuthammer (NUT)[a,b]	Habitual/customary in two connected populations (Taï North and South) and present in another (Bossou)	
Algae scoop (AE)[a,b]	Customary in one population (Bossou)	
Ground puncture (GR)[b]	Customary in one population (Goualougo), at least present in another (Fongoli)	
Seed extraction/Nut extract (SEED)[b,c,d]	Orangutans: Customary in one population (Suaq Balimbing); chimpanzees: present in two connected populations (Taï North and South)	
Marrow-pick (MA)[a,b]	Customary in two connected populations (Taï North and South), at least present in another two (Gombe, Goualougo)	High-frequency
Fluid-dip (FD)[a,b]	Customary in three populations (Taï, Lopé, Gombe), habituary in four (Assirik, Mahale K and M, Kibale)	
Ant-dip-wipe (ADW)[a]	Customary in one population (Gombe), habitual in one population (Assirik), present in one population (Bossou)	
Termite-fish leaf-midrib (TFLF)[a]	Customary in one population (Mahale K), habitual in one population (Assirik), present in one population (Bossou)	
Lever open/stick as chisel (LEV)[a-d]	Chimpanzees: customary in two populations (Gombe, Lopé), habitual in one (Taï); orangutans: rarity (rare in Ketambe and Tanjung Puting)	
Termite-fish/Tree-hole tool-use (TF)[a-d]	Chimpanzees: customary in two populations (Gombe, Mahale K), habitual in one (Assirik); orangutans: customary in one population (Suaq Balimbing)	

[a]*Behavior listed in Whiten et al. (1999, 2001) and Langergraber et al. (2011).* [b]*Behavior listed in Boesch, C. (2012).* [c]*Behavior listed in van Schaik et al. (2009).* [d]*Behavior listed in van Schaik et al. (2001).*

Material and Apparatuses

Warm-up task

Children were presented with an A4 picture of a meadow with horses and a smaller one of a farmer. The game was to help the farmer building a fence by breaking a rectangular stick of Balsa wood (l = 15 cm, b = 0.5 cm) into shorter pieces.

For pictures of the apparatuses see figure S1.

Low-frequency tasks

Insect-pound (IN). A vertical, green and opaque plastic tube (l = 10 cm, diameter = 2.2 cm) was glued to a piece of cardboard (l = 19 cm, b = 10 cm). The tool consisted of a terracotta-colored stick made of modelling clay (l = 24 cm, diameter ~ 1 cm), which had three tiny wooden spikes (l = 2 mm) at each of its ends. These were the blunt ends of skewers which had been inserted into the still wet clay. The spikes enabled the user to retrieve three balls of Play Doh® (diameter = 1 cm) from the tube by pounding and prodding them.

Perforate (PER). The apparatus consisted of two parts: A round transparent plastic box (diameter = 8 cm, h = 4 cm) containing the reward (a sticker glued to a die) and an opaque red cardboard box (14 x 11 x 6 cm) which was glued on top of the plastic box. The only entrance to the plastic box was via a hole (diameter = 4.5 cm) at the top of the upper box. There was a small slit (l = 8.5 cm, h = 1 cm) at the bottom of the cardboard box in which a round piece of flower arrangement foam (diameter = 8 cm, h ~ 0.8 cm) was inserted. This piece blocked the entrance to the plastic container. A green wooden stick (l = 19 cm, diameter = 0.6 cm) served as the tool.

Nuthammer (NUT). The anvil consisted of a cardboard (37 x 27 cm) with a soft foam surface on the left, a hard papier-mâché surface on the right (both: l = 18 cm, b = 22 cm) and a container (l = 29 cm, b = 4 cm) for the nut at the rear part of the board. Both surfaces showed a

depression (diameter ~ 7cm, h = 1.5 cm) in the middle where the nut could be placed. A sticker was placed inside a brown plastic sphere (diameter = 3.5 cm) which consisted of four equal parts made by a 3D printer and put together with a water soluble glue. A lump of clay (l = 10.5 cm, b = 5 cm) was used as the hammer.

Algae scoop (AE). A red cardboard box (30 x 23 x 11 cm) with a transparent lid and two openings in an inversed-T shape (horizontal part: l = 19 cm, h = 1 cm; vertical part: l = 3.5 cm, h = 3 cm) at the right and left side was used in this task. The box was filled with white polystyrene balls (diameter 3-5 mm) which were used as practical alternative to water. The reward consisted of a sticker attached to a black piece of light plastic foil (l = 20 cm, b = 3 cm). A wooden yellow and blue stick (l = 28 cm, diameter = 0.6 cm) served as the tool.

Ground puncture (GR). The apparatus consisted of two plastic boxes (20 x 17.5 x 12 cm) which were glued together on top of each other and covered by cardboard and colourful wrapping paper to appear like one single box (25 x 18 x 24 cm). The bottom of the upper box contained a hole (l = 10.5 cm, b = 9 cm) which was covered by a layer of blue plasticine (h = 1-1.5 cm). Two sticks inserted from the side of the box as well as a cardboard frame on top of it fixed the plasticine to the bottom of this box. The box on the bottom had round windows (diameter = 8 cm) on both long sides so that the reward inside was visible. The reward consisted of a sticker glued to a yellow piece of cardboard (l = 4 cm, b = 4 cm). A blue wooden stick (l = 51.4 cm, diameter = 2.2 cm) with a pointed end was used as the tool.

Seed extraction/Nut extract (SEED). A dark blue papier mâché box (18 x 7 x 2.5 cm) with a narrow opening at the top (l = 11 cm, b = 1 cm) was used for this task. Six pom poms (diameter = 1.5 cm) in different colors were used as the target objects which had to be removed from the box by levering. A red and blue wooden stick (l = 19 cm, diameter = 0.6 cm) served as the tool.

High-frequency tasks

Marrow pick (MA). Children were presented with a transparent test tube (l = 15 cm, diameter = 3.7 cm). The reward consisted of a sticker attached to a rolled up piece of sponge (l = 5 cm, b = 4.5 cm), which was inserted at a depth of 5.5 cm from the top (indicated by a black line). A clear Perspex stick (l = 22.7 cm, diameter = 0.5 cm) was used as a tool.

Fluid-dip (FD). The apparatus consisted of a test tube ((l = 15 cm, diameter = 3.7 cm) inserted into a yellow cardboard box (18.5 x 9 x 7 cm) and a red bottle lid (diameter = 3 cm). A small amount of yellow children's paint was used as the target object. A wooden stick (l = 19 cm, diameter = 0.6 cm) served as the tool.

Ant-dip-wipe (ADW). A transparent oval container (13 x 10.5 x 6.5 cm) with a hole (diameter = 2 cm) in the lid was glued on top of a water bottle (h = 27 cm). The bottle contained 1 liter of water and a piece of sponge with a small hole served as a lid. The oval container was filled with white polystyrene balls (diameter 3-5 mm). The apparatus was placed in a blue cardboard box (25 x 22 x 39 cm) with only 2 cm of its upper part visible. A blunt glass stick (l = 49.5 cm, diameter = 0.7 cm) was used as a tool. When removing the stick from the apparatus, the polystyrene balls would stick to its wet surface. Another object in this task was a transparent plastic container (26 x 23.5 x 12.5 cm), which children used to put the polystyrene balls in.

Termite-fish leaf-midrib (TF-LF). The apparatus consisted of a yellow cardboard box (21.5 x 9 x 12 cm) with a hole (diameter = 3.5 cm) in it. Three pieces of green sponge scourer (l = 1.5 cm, b = 1.5 cm) with a star attached to each of them were used as target objects. The tool consisted of a wooden stick (l = 28 cm) with Velcro® glued to both ends and a sturdy paper "leaf" (l = 7.5 cm, b = 7.5 cm) at 7.5 cm from one side.

Lever open/stick as chisel (LEV). A metal mug (h = 11 cm, diameter = 8 cm) covered in colorful wrapping paper was covered by a layer of blue plasticine (h = 1.5 – 2 cm) with a hole

(diameter = 0.5 – 0.7 cm) in the middle. A clay ball (diameter = 1.5 cm) with a sticker attached to it was used as the target object and placed into the mug. A yellow plastic stick (l = 13 cm) with a slightly pointed tip served as the tool.

Termite-fish/Tree-hole tool-use (TF). The apparatus consisted of a colourful sloping cardboard box (21 x 17 x 17 cm) with a hole (diameter = 3.5 cm) in it. Three pieces of green sponge scourer (l = 1.5 cm, b = 1.5 cm) with a star attached to each of them were used as target objects. The tool was built of a wooden stick (l = 28 cm) with Velcro® attached to both ends.

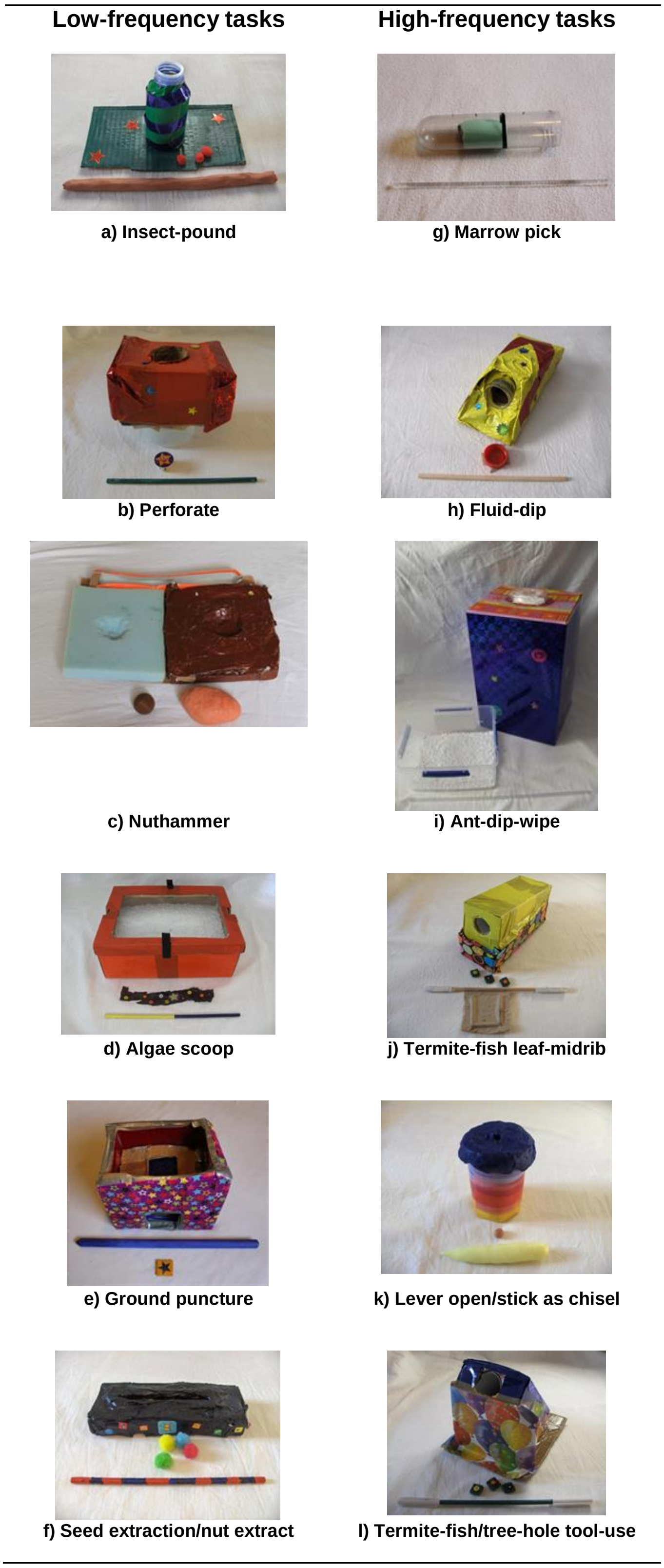

Figure S1. Apparatuses used for the GATTeB tasks. Panels 1a) to 1f) present the low-frequency tasks, panels 1g) to 1l) the high-frequency tasks.

Task instructions, procedure, and scoring

In the tool-use tasks, the experimenter (E) gave general encouragement, but never suggested using the tool. The only instructions given to the children were the task instructions stated below, and these were phrased in a fashion as general as possible, not suggesting the use of the tool as the solution to the task (e.g. in tasks Perforate, Algae scoop, Ground puncture, and Marrow pick E said “Try to get the sticker out of the box!”). The exception was Ant-dip-wipe where children were told to remove the stick from the apparatus, as in this task we were not interested in children’ spontaneous tool use, but rather on whether they spontaneously used a certain efficient strategy to remove polystyrene balls from the stick, namely holding the tool in one hand while wiping the balls off with one or more strikes of the other hand (as it is the technique in the target behavior that differs between great ape populations [s1]).

The tasks were designed as games in which children could win a sticker. As a result, the rewards involved in the studies tool behaviors differ between our human participants and the wild great apes: Whereas children’s motivation to solve the GATTeB tasks was to gain stickers, great apes in the wild engage in the respective tool behaviors in order to obtain food. Thus, the tasks are not identical with regard to the obtained reward. However, as Tomasello and Call [s2] have pointed out, what is even more important than constructing identical task contexts is establishing functionally equivalent situations and stimuli. And with this regard, our tasks are comparable to the wild great ape tool tasks: Stickers are highly motivating for most young Western children, as food is highly motivating for most wild great apes.

In six of the GATTeB tasks, the sticker was directly involved in the task, i.e. it was placed inside the apparatus and had to be retrieved with the tool. In the other six games, children were told that they could win a sticker if they solved the game (e.g. in Fluid dip, if they were able to obtain some paint from within the apparatus).

Below we present the English versions of the task instructions. The German versions can be obtained from E.R. upon request.

Warm-up task

Participants were sitting at a table or on the floor, facing E or sitting across the corner. They were presented with the picture of the meadow and the farmer and asked whether they could help the farmer building a fence. Children were then shown a stick of Balsa wood and encouraged to break it into smaller pieces. If children were hesitating, E repeated the encouragements or helped them completing the task.

E said “Before we start, I’d like to show you something! Look, here is a large meadow with some animals on it! Do you know these animals? ...What kind of animals are these? ...Yes, horses! How many horses can you see? Let’s count them! ...The horses belong to farmer Joe! Here he is! Joe wants to build a fence so that his horses cannot run away. He has already begun building the fence on this side. Do you think you can help Joe finishing the fence?...Here is a long stick and you can break this stick into smaller pieces like this one so that we can make the fence. Do you think you can do that?”

Insect-pound (IN)

E put the apparatus and the tool in front of the child. She then presented three balls of Play Doh® and put them into the tube. Children were told that they had to retrieve the balls in order to win a sticker. The tiny spikes at the end of the stick allowed the Play Doh® to stick to the tool. To count as correct tool use, children had to insert the stick into the tube and to pound the balls at the bottom of the tube. Correct success was scored when at least one of the balls was retrieved from the tube. Children had 2 min to complete this task.

E said “This is our first/next game. Here is a tube and here I have three balls of Play Doh®. Look what I do! One, two, three! If you can get the balls out again, you win a sticker!”

<u>Perforate (PER)</u>

E presented the child with the apparatus and the tool and drew his/her attention to the dice with the attached sticker in the box. They were told that they could keep the sticker if there were able to retrieve it from the box. Correct tool use was scored when children inserted the stick into the apparatus and broke the barrier of flower arrangement foam. Correct success was scored when children removed the dice from the box by tipping the apparatus. Children had 2 min to complete this task.

E said “This is our first/next game. Look, there is a sticker in the box! If you can get the sticker out of there, you can keep it!”

<u>Nuthammer (NUT)</u>

E put the apparatus, the plastic nut, and the clay hammer in front of the child and told him/her that the game was to open the ball to retrieve a sticker. Correct tool use was scored when children took the hammer in one or two hands and hit it onto the nut. Correct success was scored when children opened the plastic nut after using the hammer. Children had 2 min to complete this task.

E said “This is our first/next game. Here is a ball and there is a sticker in it. If you can get the sticker out of there, you can keep it!”

<u>Algae scoop (AE)</u>

E put the apparatus and the tool in front of the child and drew his/her attention to the sticker attached to a strip of plastic inside the box. Children were told that they could keep the

sticker if they were able to retrieve it from the apparatus. Correct tool use was scored when children inserted the stick into one of the entrances of the box and touched the strip of plastic. Correct success was scored when the strip of plastic was fully removed from the box. Children had 2 min to complete this task.

E said “This is our first/next game. Can you see the sticker? If you can get the sticker out of there, you can keep it!”

Ground puncture (GR)

Due to the size of the apparatus, the apparatus in this game was always placed on the floor. If children were sitting on a chair, E turned the chair in the direction of the apparatus. E held the box up and drew the child’s attention to the sticker which could be seen through one of the windows. Children were told they could keep the sticker if they were able to remove it from the box. Correct tool use was scored when children used the stick to make a hole in the plasticine or at least tried to puncture the plasticine layer. Correct success was scored when children made a hole and removed the sticker by putting their hand through the hole. Children had 3 min to complete this task.

E said “This is our first/next game. There’s a sticker in the box, can you see it? If you can get the sticker out of there, you can keep it!”

Seed extraction/Nut extract (SEED)

E put the apparatus and the tool in front of the child and asked whether he/she could see the pom poms inside the box. Children were then told they could win a sticker if they were able to retrieve one of the balls. Children had to use the stick in a levering fashion to push the pom poms through the small lit on top of the box. Correct tool use was scored when children inserted the tool into the apparatus and used it to lever the pom poms out of the box. Correct success was

scored when at least one of the balls was retrieved from the apparatus. Children had 2 min to complete this task.

E said “This is our first/next game. There are colourful balls in this box, can you see them? If you can get them out of there, you win a sticker!”

Marrow pick (MA)

E put the apparatus and the tool in front of the child and drew his/her attention to the sticker attached to the sponge inside the tube. Children were told they could keep the sticker if they could retrieve it from the tube. Correct tool use was scored when children inserted the stick into the tube and touched the sponge with it. Correct success was scored when children fully retrieved the sponge from the tube. Children had 1 min to complete this task.

E said “This is our first/next game. There is a sponge with a sticker in the tube. Can you see it? If you can get the sticker out of there, you can keep it!”

Fluid-dip (FD)

Children were presented with the apparatus, the bottle lid, and the stick and were then asked to have a look into the tube. They were told that there was yellow paint inside the tube and that they could win a sticker if they could get some of the paint out of the tube and into the container. Correct tool use was scored when children inserted the stick into the tube and correct success was scored when they were able to place some paint into the container. Children had 1 min to complete the task.

E said “This is our first/next game. In this box, there is something yellow! Can you see it? It is yellow paint! If you can get a little bit of the paint out of there and into here, you win a sticker!”

Ant-dip-wipe (ADW)

Due to the size of the apparatus this game always took place on the floor. E put the apparatus on the floor; the stick was already inserted in the box. If the child was sitting on a chair, E turned the chair towards the apparatus. E asked the children whether they could see the white balls inside the box and were told that they could win a sticker if they could get some of the balls out of the box and into another box next to the apparatus. Since the focus of this task was not on whether children spontaneously used the tool to accomplish the task, but on whether they used a certain efficient strategy to remove the balls from the stick, children were encouraged to pull the stick from the apparatus. Correct tool use was scored when children held the stick in one hand while wiping off the balls with the other hand – either using a close grip, the flat hand or the finger tips. Correct success was scored when children were able to remove all the balls from the stick with one of the wiping behaviors. Children had three minutes to complete this task.

E said "This is our first/next game. Can you see the white balls in the box? Do you think you can get some of these balls out of there and into this box? If you can do that, you win a sticker! ...Try to pull this stick out the box and see what happens!"

Termite-fish leaf-midrib (TFLF)

E put the apparatus and the tool in front of the child. She then presented three small pieces of sponge scourers with stars glued to them and put them into the box. Children were told they would win a sticker if they could retrieve the stars from the apparatus. The stick had Velcro® at both ends so that the stars could easily get attached to it. Attached to the stick was a paper leaf in such a way that it was impossible to reach the stars without tearing the leaf off the stick first; either end of the stick was too short to reach the stars when the leaf was still attached. Correct tool use was scored when children tore the leaf off the stick either by ripping it off with

one hand or by forcefully pounding the stick into the box and then inserted the stick into the box. Correct success was scored when children retrieved the stars with the stick after ripping off the leaf. Children had 2 min to complete this task.

E said “This is our first/next game. Here is a box and here I have three stars. Look what I do! One, two, three! If you can get the stars out of there, you can win a sticker!”

Lever open/stick as chisel (LEV)

E put the apparatus and the tool in front of the children. She then drew children’s attention to the ball in the mug by shaking the apparatus and telling children that there was a ball inside. She told the children that they could win a sticker if they were able to get the ball out of the mug. The plasticine lid of the mug already contained a small hole which children were supposed to enlarge with the tool. E pointed the hole out to the children and asked them whether they could make it larger. Correct tool use was scored when children either inserted the stick into this small hole to make it wider or when they tried to make a new hole in another place on the lid. Correct success was scored when children retrieved the ball from the mug by tipping the apparatus after they made a big hole into the lid. Children had 1 min to complete this task.

E said “This is our first/next game. There is a ball in the mug, can you hear it? There is a sticker on the ball. If you can get the ball out of there, you can keep the sticker! Look, there is already a hole in the lid. Do you think you can make it larger?”

Termite-fish/Tree-hole tool-use (TF)

E put the apparatus and the tool in front of the child. She then presented three small pieces of sponge scourers with stars glued to them and put them into the box. Children were told they would win a sticker if they could retrieve the stars from the apparatus. The stick had Velcro® at both ends so that the stars could easily get attached to the tool. Correct tool use was

scored when children inserted the stick into the box. Correct success was scored when children retrieved at least one star by using the tool. Children had 1 min to complete this task.

E said "This is our first/next game. Here is a box and here I have three stars. Look what I do! One, two, three! If you can get the stars out of there, you can win a sticker!"

Participants

Children were tested in nurseries in a large metropolitan area in the UK (*n* = 33), a Science Museum (*n* = 9) and our Infant and Child Laboratory (*n* = 3) in this city as well as in a nursery in a small town in southern Germany (*n* = 5). The ethnic background of the sample was mostly Caucasian (68%), 26% of the children were Black and 6% Asian. Children in nurseries were recruited via information letters sent to the parents after an initial contact with the nurseries had been established. Those tested at the Science Museum were either recruited via an advertisement on the museum website or parents were approached directly in the museum. Children tested in the laboratory were recruited via an existing database. The testing situation was comparable across the testing sites: The experiment took place on the floor in a separate room or quiet corner of a room in the nurseries or the science museum. Children were always tested individually by the same female experimenter, and all received the same warm-up procedure by the experimenter. An additional 22 children were tested but had to be excluded from the analysis because they were too shy (*n* = 2), cried (*n* = 1) or did not match the required age range (*n* = 19. The children who did not match the age range were all older (up to 52 months), but were tested as in some nurseries information on children's age was given to us only after data collection was completed).

2. Supplementary Data

Fifty children completed a set of four tasks each, resulting in 200 trials of which 193 were valid. One trial had to be excluded because of an intervention of nursery staff, two trials due to experimenter error. Four trials were excluded after being scored as incorrect success: In NUT, one child succeeded by pounding the ball directly on the ground. Another child managed to open the nut by tearing it apart with his fingers. In AE, one subject was able to insert his hand through one of the openings of the apparatus and to extract the target object. Similarly, in PER, one child managed to put her hand in the apparatus and to break the barrier with her fingers.

In order to be able to conclude whether a given tool behavior was within the spontaneous capacities of our participants, we required to observe the spontaneous invention of the behavior in at least two participants. Positive evidence from a single child was regarded as insufficient because the observed action could have been produced by chance. While of course it would still be possible that two children showing the correct tool-use behavior could both have produced the behavior by chance, we think this is highly unlikely. There is always a (small) likelihood that a random behavior produced by a given participant matches the target behavior by chance, i.e. the correct tool behavior in the given task. Yet, this chance is likely to be small in every case because generating the correct solution in any of the GATTeB tasks requires a behavioral sequence consisting of several steps (e.g. in Insect pound: pick up stick – insert stick in tube – prod play doh with stick – extract stick). Whereas single elements may indeed be produced or supported by chance, the likelihood that the sum of these elements is generated by chance becomes very small (for a similar argument see s3).Conservatively, we still acknowledge that this may possibly happen in one subject per condition by chance alone. But the chance likelihoods become even smaller when we would have to assume more than one subject to produce the behavior by mere chance alone. This is because in such a case we would have to multiply small likelihoods with each other, which produces likelihoods that are exceedingly

small. This allows us to exclude chance as a feasible explanation for double occurrences of a given behavior (but, again: not for single occurrences).

Our results indicate that on average, great ape low-frequency tasks were more difficult to solve for children and that great ape high-frequency tasks were easier to solve for our participants. Therefore, we found that overall there was a match in frequency categories between apes and children. However, we acknowledge that this match was not perfect. For example, some tasks in the low-frequency group had rather high success rates (see e.g. Algae scoop), whereas some tasks in the high-frequency group seemed unusually difficult for the children (e.g. Lever open). Future studies might benefit from this fact as it might be worthwhile to look into detail why some of the tasks did not match this pattern.

We found that for both low- and high-frequency tasks the majority of successful children (78% in the low-frequency tasks, 70% in the high-frequency tasks) were able to solve the tasks immediately, i.e. they chose to use the tool as their very first attempt to solve the task (i.e. they did not use other strategies such as using their hands before they chose to use the tool) and once they picked up the tool, they solved the task "in one go", i.e. without a change of strategies or setting aside the tool in between. Thus, we do not believe that children's success in the tasks can solely be accounted for by individual trial-and error learning.

Each child only received one trial per task, Therefore, our approach to studying whether young children would be able to spontaneously invent the necessary tool-use behaviors was rather conservative. Consequently, we do not know whether a child producing a correct tool behavior in a given task would also be able to reproduce this behavior on following trials or whether the behavior in the first trial occurred only by chance and without insight. Implementing more than one trial would grant children with more time and opportunities to learn the correct behavior individually. In the current study, this would have been especially interesting with

regard to the Nuthammer, in which only one child produced the correct tool behavior. Would this child be able to use the tool correctly on the following trial, and maybe even be successful? Future studies administering several trials per GATTeB task are needed to address questions like these.

In the following, we will give a more detailed description of our statistical analysis. We investigated whether low- and high-frequency tasks differed with regard to their rates of tool pickup/use, correct tool use, and correct success by using Generalized Linear Mixed Models (GLMM) with a binomial error structure and a logit link function in R version 3.0.2 using the glmer function of the R package lme4 [s4]. The aim was to find three models (one for each dependent variable) which would explain the data best given the predictors age, sex, and frequency. We started by specifying full models including the maximal number of fixed and random effects: Sex, age, and frequency were entered as fixed effects (we also entered an interaction term between age and frequency; as it was not significant, we only report the models with the main effects here). The random effects structure was: A random intercept for subjects (to account for the fact that data points were not independent of each other) and a random slope for frequency on subjects, with subjects being nested within the variable *nursery.* The random slope allowed children to respond differently to high- vs. low-frequency tasks. The random intercept and the random slope were allowed to covary.

We used a backward elimination procedure using the “drop1” function to derive the most parsimonious models with the best model fit. We systematically dropped terms that did not significantly contribute to the model fit, i.e. whose removal did not lead to a significantly worse model fit, a change in the significance level of the predictors or a change in the Odds Ratios (OR) of the predictors greater than 10%. We made sure that the models always consisted of the same random effects structure at each step in the elimination process. I.e., if we had to remove a

random effect in the equation for one of the outcome variables – e.g. because of non-convergence of the model – we did the same for the other two dependent variables.

For each model we computed p-values, OR, and Confidence Intervals (CI) for the individual predictors. Maximum Likelihood tests were used to derive p-values of the predictors by comparing models including the respective factor with those not including them (using the R function "anova" with the argument "test" set to "Chisq"). Model stability of the three final models was determined by a comparison of the estimates of a model based on the complete data set with those derived from models where the levels of the random factor subject were excluded one at a time. To investigate possible problems concerning multicollinearity, we calculated a standard linear model of the final model which excluded the random effects and determined the Variance Inflation Factor (VIF). We looked for potentially influential cases by using the "DFBETAS" function of the R package "influence.ME".

GLMM: Tool Pickup/Use

Since tool pickup/use was not meaningful for ADW (children were told to pick up the tool), we excluded this task for the according GLMMs, resulting in 176 valid trials for these analyses. The initial full model estimated the parameter for frequency with only low fidelity, indicated by a large OR (169) and a wide corresponding CI between 5 and 5451. Therefore, a simplification of the random effects structure was necessary: Excluding the correlation between the random factors, dropping nursery or doing both did not result in a more stable model. Only the exclusion of the random slope for frequency did so. The resulting full model was not able to explain the data better than a null model consisting of only the random effects structure ($\chi^2(3)$ = 2.363, p =.501).

The "drop1" function suggested that no predictor contributed significantly to the model fit. Since the removal of sex suggested the greatest reduction in the AIC and we did not find any

gender effects in the other models, we decided to drop this variable. This did not lead to changes in the significance levels of the predictors and changes in the OR were smaller than 1%.

Although the "drop1" function suggested that age did not significantly contribute to the model fit either, it remained in the equation to allow comparison with the model for correct success, for which the effect of age was significant. Finally, nursery was removed since the variance it accounted for was small. This did not result in any changes in the significance levels or OR of the predictors. This final model was still not able to explain the data significantly better than a null model only consisting of the random effects structure ($\chi^2(1) = 2.362$, $p = .124$). The model revealed that frequency did not have an effect on children's rates of tool pick up/use (p = .376).

We found the model to be fairly stable with regard to frequency and age. There were no problems with multicollinearity in the model (VIFs for both frequency and trial were 1.000). We found nine influential observations. An exclusion of these data points and a recalculation of the model did not lead to a change of the significance levels for frequency. The change in the OR for age was smaller than 1%, the OR for frequency increased by 13%.

GLMM: Correct Tool use

Since we had to drop the random slope for frequency in the model for Tool pickup/use, we did the same for the model for Correct tool use in order to ensure comparability. The simplified full model was not able to predict the data better than a null model consisting only of the random effects structure ($\chi^2(3) = 2.432$, $p = .488$).

The "drop1" function suggested that no predictor contributed significantly to the model fit. Since the removal of sex suggested the greatest reduction in the AIC and we did not find any gender effects in the other models, we decided to drop this variable. There were no changes in the significance level of the predictors and the changes in the OR were smaller than 1%.

Finally, nursery was removed since the variance it accounted for was extremely small. Compared to a null model only consisting of the same random effects structure, the final model was not able to explain the data significantly better ($\chi^2(2) = 4.142$, p =.126). The model revealed that frequency did not have an effect on children's probability of correctly using the tool (p = .260).

We found this model to be very stable with regard to frequency and age. There were no problems with multicollinearity in the model: The values were 1.000 for both frequency and age. We found 14 potentially influential observations. An exclusion of these data points and a recalculation of the model did not lead to changes in the significance levels of the predictors. The change in the OR for age was smaller than 1%, the OR for frequency changed by about 14%.

GLMM: Correct success

Since we had to drop the random slope for frequency in the model for Tool pickup/use, we did the same for this model to ensure comparability across the dependent variables. The simplified full model predicted the data better than a null model consisting only of the random effects structure ($\chi^2(3) = 32.217$, $p < .001$).

The "drop1" function suggested that sex did not contribute significantly to the model fit, so it was dropped from the model. This did not lead to any changes in the significance level or OR of the predictors.

Finally, we decided to drop nursery since the variance it accounted for was extremely small. There were no changes in the significance levels or OR of the predictors. The final model was able to explain the data significantly better than a null model just comprising the random effects structure ($\chi^2(2) = 33.377$, $p < .001$). The model revealed a significant positive effect of age ($p < .001$): With each month increase in age, children were 1.3 (95% confidence interval (CI) [1.1; 1.4]) times more likely to succeed. On top of this age effect, frequency was a significant

predictor for correct success (p < .001): Compared to low-frequency tasks, tasks in the high-frequency group were 4.4 (95% CI [2.1, 9.1]) times more likely to be solved (Table s3).

Table S3. Final Generalized Linear Mixed Model for variable correct success.

Term	X^2	df	p	OR	CI-OR_{lower}	CI-OR_{upper}
Intercept	-	-	-	3.677e-05	4.962e-07	0.003
Age	16.646	1	<.001	1.289	1.139	1.459
Frequency	16.521	1	<.001	4.398	2.124	9.104

Number of observations: 193. OR = Odds ratio; CI = Confidence interval

We found this model to be very stable with regard to age and frequency. The VIFs for age and frequency were 1.000, indicating no problems with multicollinearity in the model. We looked for potentially influential cases by using the DFBETAS function of the R package ‘influence.ME’ and found 16 observations above the cut-off value. An exclusion of these data points and a recalculation of the model did not result changes of the significance levels of the predictors. The changes in the OR of the predictors were smaller than 6%.

Analysis of factor time

In order to investigate whether children’s performance was affected by the fact that tasks were allocated differing amount of times for their completion (1, 2 or 3 min), we reran the models including a fixed effect for time. We found that time did not make any significant contribution in the model for tool pickup/use ($\chi^2(1) = 0.555$, p = .456). In the model for correct

tool use, time had a significant negative effect on children's behavior ($\chi^2(1) = 6.338$, $p = .012$). With every minute increase in the time allocated to the tasks, children were 0.4 (95% CI [0.2; 0.8] times less likely to spontaneously use the tools in the correct way. This means that in those tasks for which we chose to allocate more time, children were less likely to show the correct tool use. This reflects the very reason why we decided to allow children a longer time span for some of the tasks: In a pilot study, where we initially administered only one minute across all tasks, some tasks appeared to be harder for children as correct tool use was observed less often. Thus, we decided to extend the time for these tasks. The results thus reflect that there was a correlation between task difficulty and solution time.

Adult study

In order to rule out the possibility that the difference in performance between the low- and the high-frequency tasks could be explained by evident differences in the task design or difficulty, we conducted a small post-hoc study in which we presented twelve adult participants with the test battery and asked them to classify the tasks into two groups containing six tasks each. We then scored the number of "correctly" grouped tasks (minimum: 3; maximum: 6). Thereby we were able to investigate whether subjects would be able to reproduce the classification into low- and high-frequency tasks. Having categorized the tasks, participants were asked to name the criteria they used to split the tasks into two groups.

The results show that three participants grouped three tasks correctly, seven subjects had four tasks correct, and two people classified five tasks correctly. The average number of correctly classified tasks was 3.92 (SD = 0.67). We used a Chi-square goodness-of-fit test to compare the distribution of subjects' responses with a distribution resulting from chance classification (expected values drawn from a simulated sample with $n = 100{,}000$) and found that subjects were significantly better than expected by chance (χ^2 (2) = 20.35, $p < .001$). This is not surprising given the fact that the tasks possess some well-perceptible task-inherent similarities

which can be used as points of reference for categorization. This is also reflected by the criteria subjects used: Most of the criteria were related to whether the task involved breaking/destruction of objects (e.g. NUT, LEV, GR), obtaining the target object via extraction (e.g. AE, TF, MA) or whether the target object was visible (e.g. MA, GR, AE).

Most critically however, no participant created a classification equal to the low- and high-frequency split. Furthermore, only one participant mentioned difficulty as a criterion and was therewith able to classify four low-frequency tasks correctly (NUT, GR, AE, IN). However, she claimed that the group containing these four tasks was the easier one, thus not matching the results we found with the children. Thus, our overall conclusion is that although some tasks share some apparent features, these features are not able to distinguish between the low- and high-frequency tasks and thus cannot account for our finding that low- and high-frequency tasks differ in their success rates.

3. Supplementary References

s1. Schöning C, Humle T, Möbius Y, McGrew WC. 2008. The nature of culture: Technological variation in chimpanzee predation on army ants revisited. *J. Hum. Evol.* **55**, 48-59. (doi:10.1016/j.jhevol.2007.12.002.)

s2. Tomasello M, Call J. 2008. Assessing the validity of ape-human comparisons: A reply to boesch (2007). *J. Comp. Psych.* **122**, 449-452.

s3. Koehler W. 1925. *The mentality of apes,* 2nd edn. Liveright, US.

s4. Bates D, Maechler M, Bolker B, Walker S. 2013. *lme4: Linear mixed-effects models using Eigen and S4.* R package version 1.1-6.
